# First generation of multifunctional peptides derived from latarcin-3a from *Lachesana tarabaevi* spider toxin

**DOI:** 10.3389/fmicb.2022.965621

**Published:** 2022-09-21

**Authors:** Luiz Filipe Ramalho Nunes de Moraes, Patrícia Souza e Silva, Tábata Camila Pereira Leite Pereira, Thiago Antônio Almeida Rodrigues, Breno Emanuel Farias Frihling, Rosiane Andrade da Costa, Heron Fernandes Vieira Torquato, Cauê Santos Lima, Edgar Julian Paredes-Gamero, Ludovico Migliolo

**Affiliations:** ^1^S-Inova Biotech, Programa de Pós-Graduação em Biotecnologia, Universidade Católica Dom Bosco, Campo Grande, MS, Brazil; ^2^Selkis Biotech, Startup, Laboratório de Artrópodes Peçonhentos, Campo Grande, MS, Brazil; ^3^Programa de Pós-Graduação em Ciências Genômica e Biotecnologia, Universidade Católica de Brasília, Brasília, DF, Brazil; ^4^Programa de Pós-Graduação em Biotecnologia, Universidade Federal de Mato Grosso do Sul, Campo Grande, MS, Brazil; ^5^Programa de Pós-Graduação em Bioquímica, Universidade Federal do Rio Grande do Norte, Natal, RN, Brazil

**Keywords:** spider, antimicrobial peptides, resistant bacteria, antitumor, drug design

## Abstract

The need for discovering new compounds that can act selectively on pathogens is becoming increasingly evident, given the number of deaths worldwide due to bacterial infections or tumor cells. New multifunctional biotechnological tools are being sought, including compounds present in spider venoms, which have high biotechnological potential. The present work aims to perform the rational design and functional evaluation of synthetic peptides derived from *Lachesana tarabaevi* spider toxin, known as latarcin-3a. The antimicrobial activity was tested against Gram-positive and -negative bacteria, with minimum inhibitory concentrations (MIC) between 4 and 128 μg.ml^−1^. Anti-biofilm tests were then performed to obtain MICs, where the peptides demonstrated activity from 4 to 128 μg.ml^−1^. *In vitro* cell cytotoxicity assays were carried out from tumor cell lines, lineages C1498, Kasumi-1, K-562, Jurkat, MOLT4, and Raji. Erythrocyte integrity was evaluated in the presence of synthetic peptides analog, which did not promote hemolysis at 128 μg.ml^−1^. The peptide that showed the best antibacterial activity was Lt-MAP3 and the best antitumor was Lt-MAP2. In conclusion, rational design of multifunctional antimicrobial peptides may be promising alternative tools in the treatment of emerging diseases such as bacterial infections and tumor cells.

## Introduction

Bacterial infections and tumors are among the leading causes of death around the world (Glaziou et al., 2018; Mattiuzzi and Lippi, 2019). Currently, with the emergence of antibacterial resistance and increased exposure to carcinogens, the mortality rate from these diseases has increased (Zhu et al., 2017). Resistance to medicines used in the treatment of bacteria and tumor cells is a worldwide problem that can lead to worse patient outcomes or may prolong the disease (Munita and Arias, 2016; Bukowski et al., 2020).

Among types of antibacterial resistance, biofilm formation is responsible for prolonging the infection in numerous clinical cases, such as in catheter application, intubation, prosthesis, and post-surgery, among others (del Pozo, 2018). Biofilm consists of aggregated planktonic cells that assume multicellular and coordinated development, involving the life cycles and maturation of one or more bacterial species encapsulated in a carbohydrate matrix that generally prevents antibiotics from entering and thus contributing to bacterial resistance to administered medicines (Rumbaugh and Sauer, 2020). Furthermore, multidrug-resistant bacteria are increasingly less susceptible to conventional antibiotics, and cases of resistant bacterial infections are becoming more common (Agyepong et al., 2018).

Another big problem are tumors, which are cell clones with a high proliferation rate and which lack regulatory mechanisms for growth suppression, presenting a similar challenge in a different context. When there is a lack of an appropriate treatment or specific medicine for a given tumor type, this can result in a high cell proliferation rate, making the tumor invasive and leading patients to death (Deslouches and Di, 2017).

Among the various tumor types, one of the most malignant is leukemia, and its appearance is more common in childhood, with children accounting for 30% of the cases. Leukemias can be classified into acute lymphoblastic leukemia, acute myeloid leukemia, chronic lymphoid leukemia, chronic myelomonocytic leukemia, or chronic myeloid leukemia, whereas acute lymphoblastic leukemia is the common type (Kaplan, 2019).

In the last decade, medicine related to tumor treatment have undergone several advances, however, this disease remains a serious threat to human survival (Hashim et al., 2016). The use of chemotherapy in conjunction with surgery and radiotherapy has revolutionized the treatment, promoting an increase in the tumor patient’s life expectancy (Bratt et al., 2015).

However, anti-tumor treatment techniques can be considered evasive and non-selective, also reaching healthy cells, resulting in side effects and cytotoxicity, contributing to resistance development by tumor cells (Chatterjee et al., 2014; Franzoi et al., 2021). Thus, an alternative method is the discovery of new medicine classes that would act on tumor cells, without or with low toxicity to normal cells (Deslouches and Di, 2017).

In addition, other peptides derived from invertebrate animal toxins also are commercially used, such as honeybee toxin (Apitox®, Uruguay) used as a cosmetic compound from *Apis mellifera*; bivalirudin (Angiomax®, United States) used for thrombin inhibitor anticoagulant agents from leech *Hirudo medicinalis*; and ziconotide (Prialt®, Brazil) used to treat chronic pain, derived from mollusk *Conus magus* (Bordon et al., 2020).

Antimicrobial peptides (AMPs) in general are small molecules, cationic, amphipathic α-helical structure, and have specific or multiple activities targets, such as antifungal, insecticidal, antiviral, antibacterial, and antitumor action (Izadpanah and Gallo, 2005; Pfalzgraff et al., 2018).

As an example, studies have been carried out on natural peptides from spider toxins, such as lycotoxin-I and lycotoxin-II from *Lycosa carolinensis* and latarcins and latartoxins from *Lachesana tarabaevi* (Wang and Wang, 2016). The Lycosidae family spiders are the most studied with their toxic activities already well characterized, being amphipathic peptides mostly α-helical structured, presenting antibacterial, antitumor, antifungal and insecticidal activities (Braga et al., 2020).

The latarcin-3a (Ltc-3a), isolated of spider venoms from *L. tarabaevi* has demonstrated activity against *Arthrobacter globiformis*, *Bacillus subtilis*, *Escherichia coli* DH5-alpha, and *E. coli* DH1. It also showed antifungal activity against *Pichia pastoris* and *Saccharomyces cerevisiae* (Kozlov et al., 2006).

Peptides have distinct functions, such as latarcin toxins that act non-selectively as cytolytic peptides for extracorporeal digestion process, proposing a “carpet” model-like mechanism of action (Kozlov et al., 2006; Dubovskii et al., 2008). Currently, studies with other latarcins including Ltc-3a from *L. tarabaevi* have demonstrated better antibacterial properties, through inhibition of *E. coli* ATP synthase with amidated C-terminal (Syed et al., 2018). In addition, the lycosin-I peptide from *Lycosa singorensis* also demonstrated the same mechanism of action on *E. coli* ATP synthase with amidated and carboxylated C-terminal (Amini et al., 2020).

Based on this, rationally designed peptide analogs with variations in physical–chemical parameters, such as charge, hydrophobicity, polypeptide chain length, and amino acid composition, can help to understand the mechanisms of interaction with specific pathogens, providing the peptides with multifunctional activities (Migliolo et al., 2012).

In view of the alarming growth of bacterial resistance and the risk that hospital infections represent for the world population, as well as the growing number of tumor cases, especially leukemias, an initial study of efficient and less invasive alternatives is necessary (Elsland and Neefjes, 2018). These protein compounds play an important role in the innate immunity of the host, making the peptides targets of studies for the development of multifunctional drugs (Yeung et al., 2011; Cervelló et al., 2022). The present work aims to construct bioinspired synthetic peptide analogs based on Ltc-3a against planktonic, and sessile pathogenic bacteria, besides hemocytes and leukemia tumoral cell lineages.

## Materials and methods

### Rational design

The α-helix region of 13 amino acid residues observed in the parent peptide Ltc-3a (NH_2_-MAKKLKEYMEKLK-COOH) was used as a model for the generation of the analogs. The region comprises 5–17 amino acid residues and was chosen for the physical–chemical modifications. Aiming to optimize the analogs, the helical wheel diagram was used to promote a rearrangement of hydrophilic and hydrophobic residues. The projections were built using the HeliQuest server (http://heliquest.ipmc.cnrs.fr/; Gautier et al., 2008). The ClustalW server^1^ was used to perform the analog sequence alignment (Thompson et al., 1994). To calculate the physicochemical properties, such as charge, hydrophobicity, and molecular mass, the Antimicrobial Peptide Database (APD) prediction tool was used (http://aps.unmc.edu/AP/main.php; Wang et al., 2016). The generation consists of three new peptides (Lt-MAP1, Lt-MAP2, and Lt-MAP3) that underwent reorganization of the hydrophilic and hydrophobic face, with two partial or total substitutions of alanines to leucines and glutamic acids to lysines, varying according to the analog. In addition, all peptide analogs maintained the maximum 30% of difference (approximately three residues modified) in the amino acid composition, when compared to the parent sequence (Livingstone and Barton, 1993).

### *In silico* molecular modeling

The method was performed by the server I-TASSER^2^ using the comparative modeling method of the threading type, based on the hierarchical approach for predicting three-dimensional structures using templates obtained by aligning sequences deposited in the Protein Data Bank (PDBl https://www.rcsb.org/). The atomic models were built from iterative simulations, from an assembly of fragments based on templates (Yang et al., 2014). The validation of the models was performed based on the statistical data, expressed as C-score, Z-score, and root-mean-square deviation (RMSD) generated by the I-TASSER server. The results of the three-dimensional structures were visualized in the PyMol viewer version 2.3 (https://pymol.org/2/; DeLano, 2002).

### Peptide synthesis and purification

The peptides were synthesized by the stepwise solid-phase method using the N-9-fluorenylmethyloxycarbonyl (Fmoc) strategy with a Rink amide resin (0.52 mmol.g^−1^; Merrifield, 1986). Couplings were performed with 1,3-diisopropylcarbodiimide/ 1-hydroxybenzotriazole (DIC/ HOBt) in N,N-dimethylformamide (DMF) for 60–120 min. Fmoc deprotections (15 min, twice) were conducted with 4-methylpiperidine:DMF solution (1∶4, by volume). Cleavage from the resin and final deprotection of side chains were performed with trifluoroacetic acid (TFA):water:1,2-ethanedithiol (EDT):triisopropylsilane (TIS), 94.0∶2.5∶2.5∶1.0, by volume, at room temperature for 90 min. After this, the crude product was precipitated with cold diisopropyl ether, collected by filtration and solubilized in 200 ml aqueous acetonitrile at 50% (by volume). The extracted peptide was twice freeze-dried for purification. Amino acid derivatives and other reagents for the solid-phase peptide synthesis were from Merck-Novabiochem (Whitehouse Station, NJ, United States), from Peptides International (Louisville, KY, United States), or from Sigma-Aldrich (St Louis, MO, United States).

For the next step, the peptides were purified by high performance liquid chromatography (HPLC) on a reversed-phase column RP-C18 4.6 mm × 250 mm in a flow of 1 ml.min^−1^, using the solvents (phase A = 0.1% TFA:H_2_O, phase B = CH_3_CN:H_2_O 9:1 with TFA 0.1%) and the detector was at 220 nm in a gradient from 5 to 100% in 25 min by Aminothec Company (Sorocaba, Brazil). All synthetic peptides after purification were lyophilized and then solubilized in ultra-pure water for *in vitro* assays.

### Peptide sequencing

The sequences of the synthetic peptides were confirmed by Matrix Assisted Laser Desorption Ionization Time of Flight Mass Spectrometry (MALDI-ToF-MS; Pinto et al., 2016). The peptides were resuspended in ultra-pure water and mixed with saturated solution of α-cyano-4-hydroxycinnamic acid (10 mg.ml^−1^ in 50% acetonitrile, 0.1% trifluoroacetic acid) in the ratio of 1:3 (v:v) and then directly applied onto a massive plate. The samples were dried at room temperature and the monoisotopic mass spectra were acquired in reflected mode with a range of 700–3,500 m.z^−1^ with external calibration, and the MS/MS spectra were acquired using the MS/MS LIFT method (MALDI-TOF; Autoflex Speed, Bruker Daltonics, Bremen, Germany). The primary sequence of all synthetic peptides was determined manually using FlexAnalysis 3.3 software (Bruker Daltonics).

### Minimum inhibitory concentration and minimal bactericidal concentration

The minimum inhibitory concentration MIC assays were performed with non-resistant (American Type Culture Collection—ATCC) and resistant (*Klebsiella pneumoniae* Carbapenemase—KPC^+^) strains of *Acinetobacter baumannii* (003324845—clinical isolate, Hospital Regional Asa Norte, Brasília), *E. coli* (ATCC 25922) and *E. coli* (KPC 001812446—clinical isolate, LACEN, Brasília), *K. pneumoniae* (ATCC 13883) and *K. pneumoniae* (KPC 001450421—clinical isolate, LACEN, Brasília), *Pseudomonas aeruginosa* (KPC 003321199—clinical isolate, LACEN, Brasília), *Propionibacterium acnes* (ATCC 51277), and *Staphylococcus aureus* (7133623—clinical isolate). The bacteria were plated on Mueller-Hinton-agar (MHA) plates and incubated at 37°C overnight. After that period, three colonies isolated from each bacterium were inoculated in 5 ml of Mueller-Hinton-broth (MHB) and incubated at 200 rpm, at 37°C, overnight. Bacterial growth was monitored by a spectrophotometer at 600 nm. MIC tests were performed using the 96-well microplate dilution method at the final bacterial concentration of 2–5 × 10^5^ CFU.ml^−1^, as previously described in Clinical and Laboratory Standards Institute (CLSI, 2020, M100). The peptides were tested in concentrations ranging from 4 to 128 μg.ml^−1^. Ciprofloxacin was used as a positive control at the same concentrations as the peptides, while the bacterial suspension in MHB was used as a negative control. The microplates were incubated at 37°C for 18 h, and the readings were taken in a microplate Multiskan Go (Thermo Scientific) at 600 nm after the incubation time. MIC was determined to be the lowest concentration of peptide in which there was no significant bacterial growth. The replicates of 10 μl were taken from the microplate wells, plated on MHA, and incubated at 37°C for 24 h. The minimal bactericidal concentration (MBC) was determined as the lowest concentration of peptide in which no bacterial growth was detected. All experiments were carried out in biological and technical triplicates.

### Minimal biofilm inhibitory concentration

Biofilm formation was obtained using Basal Medium 2 [BM2; 62 mM potassium phosphate, 7 mM (NH_4_) 2 SO_4_, 2 mM MgSO_4_, 10 μM FeSO_4_, and 0.4% glucose]. The bacteria *A. baumannii* (ATCC 001121216) and *E. coli* (ATCC 25922) were cultured for 18 h in MHB, after which cultures were diluted 1:100 (v:v) in BM2, and the bacterial suspensions were plated in 96-well round bottom plates containing the peptides in serial dilutions of 4–128 μg.ml^−1^. The microplates were incubated for 24 h at 37°C. Negative growth control contained only bacteria, whereas ciprofloxacin was used as the positive control at the same concentrations as the peptides. Planktonic cell growth was assessed using absorbance at 600 nm. To evaluate biofilm formation, the medium was removed from the microplates and the wells were washed twice with deionized water. Adherent cells were stained with 0.01% crystal violet for 20 min. Further, the wells of the microplate were washed twice with deionized water and air-dried, and the crystal violet adhered to cells was solubilized with 110 μl of 60% ethanol. Biofilm formation was measured using absorbance at 595 nm. All absorbance readings were performed with Multiskan Go (Thermo Scientific; Silva et al., 2022). All experiments were carried out in biological and technical triplicate.

### *In vitro* hemolytic assay

The erythrocytes from Swiss mice *Mus musculus* were washed three times with 50 mM phosphate buffer saline (PBS), pH 7.4. The peptide solutions were added to the erythrocyte suspension (1% by volume) in a final concentration ranging from 4 to 128 μg.ml^−1^. Samples were incubated at room temperature for 60 min. Hemoglobin release was monitored by measuring the absorbance from the supernatant at 415 nm, using Multiskan Go (Thermo Scientific; Silva et al., 2022). Zero hemolysis (blank) was determined with suspended erythrocytes in the presence of 50 μM PBS, pH 7.4, whereas an aqueous solution of 1% (by volume) triton X-100 was used as a positive control (100% lysis of erythrocytes). This experiment was approved by the Ethics Committee (CEUA) of the Dom Bosco Catholic University under number 014/2018. Hemolytic assays were performed in triplicate.

### Cells lines

For cell assays, human and murine leukemia cell lines were used; C1498 (murine myeloid leukemia), Jurkat (human acute T-cell leukemia), K-562 (chronic myeloid leukemia), Kasumi-1 (acute human myeloid leukemia), Raji (Burkitt’s lymphoma), and MOLT-4 (acute lymphoblastic leukemia), all were purchased from the Rio de Janeiro cell bank (BCRJ). After thawing, the cells were maintained in the Roswell Park Memorial Institute (RPMI) 1,640 culture medium. All strains were supplemented with 10% fetal bovine serum, 100 μg.ml^−1^ of penicillin and 100 μg.ml^−1^ of streptomycin and subcultured every 2 or 3 days. The strains, as cells in suspension, were removed from the culture flasks only with inversion in falcon tubes, then centrifuged, counted, and resuspended in a concentration of 10^5^ cell.ml^−1^ in new culture medium and returned to the culture flasks. The cells were cultured in culture flasks and kept in an oven at 37°C and an atmosphere of 5% CO_2_.

### Metabolic activity assessment

Cellular tests for screening and constructing concentration/ response curves of the peptides Ltc-3a and analogs Lt-MAP1, Lt-MAP2, and Lt-MAP3 were performed with resazurin dye (Präbst et al., 2017). The pre-selection of the compounds was carried out after 72 h of treatment and tested in a single concentration, 50 μM, in order to find the most promising peptides for further testing. Five thousand cells of the strains were seeded in 96-well plates in a final volume of 100 μl (10^5^ cells.ml^−1^). The substances that produced approximately 60% of cell death were considered active. These criteria were adopted and adapted, based on the procedures of the National Cancer Institute (NCI-USA) in the investigation of natural substances with antitumor potential (Monks et al., 1991).

For the construction of the concentration/response curves and determination of the EC_50_, 24 h experiments were performed. Cells (10^5^ cells.ml^−1^) from lines C1498 and K562 were seeded in 96-well plates in a final volume of 100 μl, and treated with the compounds (50, 25, 12.5, 6.25, and 3.12 μM). For reading the experiments at 24 and 72 h, a new culture medium with 5% resazurin was added to the wells, after removing the culture medium with the treatments. The cells were incubated for 4 h in an oven at 37°C. After the period, the 96-well plate were read on the FlexStation 3 microplate reader (Molecular Devices) with wavelengths of 560 and 590 nm of absorbance.

### Statistical analysis

The statistical significance of the experimental results was determined by one-way Student’s *t*-test or one-way ANOVA followed by Dunnett’s test. Values of *p* > 0.01 and 0.001 were considered statistically significant. GraphPad Prism version 8.0 was used for all statistical analyses.

## Results

### Synthesis, purification, and mass spectrometry of the peptides

Ltc-3a (NH_2_-SWKSMAKKLKEYMEKLKQRA-COOH) is a non-selective cytolytic peptide, which has a α-helical structure with a hydrophobic moment of 0.575, +6 net charge, and 35% of hydrophobicity, with antimicrobial activity. For the new peptides, the region (^5^MAKKLKEYMEKLK^17^) of 13 amino acid residues, with +3 net charge and 38% of hydrophobicity and 0.699 of hydrophobic moment was selected due to the physicochemical characteristics and the α-helix conformation. The rational design intention was to increase the total net charge, adjust the hydrophobicity and reorganize the hydrophobic moment, keeping the α-helix. The new peptides were denominated Lt-MAPs, (Lt-MAP1, Lt-MAP2, and Lt-MAP3) due to the multiple activity observed.

For the first generation, Lt-MAP1, the amino acid residues at positions Met^5^, Met^13^, and Lys^17^ were replaced by Leu, Leu, and Val (NH_2_-LAKKLKEYLEKLV-COOH), allowing a reduction of the net charge to +2, conserving 35% of hydrophobicity, with amphipathicity organized with a hydrophobic moment of 0.740. For Lt-MAP2, residues Met^5^, Ala^6^, Met^13^, Glu^14^, and Lys^17^ were replaced by Leu, Ile, Leu, Lys, and Ile (NH_2_-LIKKLKEYLKKLI-COOH), promoting a net charge +4 and an increase in hydrophobicity to 46%, with the hydrophobic moment of 0.839. Lt-MAP3 shows modification in the residues Met^5^, Lys^10^, Glu^11^, Met^13^, Glu^14^, Leu^16^, and Lys^17^ for Leu, Ala, Lys, Leu, Lys, Ala, and Leu (NH_2_-LAKKLAKYLKKAL-COOH), with +5 net charge, increasing hydrophobicity to 53% and its hydrophobic moment to 0.701 (Table 1).

**Table 1:** Sequence of the parental peptide Ltc-3a and the analogs (Lt-MAP1, Lt-MAP2, and Lt-MAP3) with their emphasized physicochemical properties.

Peptide	Sequence	Z	H	H%	µH	M (Da)	M* (Da)
Ltc-3a	SWKSMAKKLKEYMEKLKQRA	6	0.058	35	0.575	2484.0	2483.3
Lt-MAP1	LAKKLKEYLEKLV	2	0.312	46	0.740	1575.0	1575.2
Lt-MAP2	LIKKLKEYLKKLI	4	0.444	46	0.839	1630.2	1630.2
Lt-MAP3	LAKKLAKYLKKAL	5	0.288	53	0.701	1488.0	1488.0

Of which <Z>: net charge; <H>: Hydrophobicity; H%: percentage of non-polar residues; <μH>: hydrophobic moment; M: Theoretical mass determined by the Antimicrobial Peptide Database prediction tool; M*: actual mass determined by mass spectrometry after synthesis and purification of peptides

**Figure 1:**
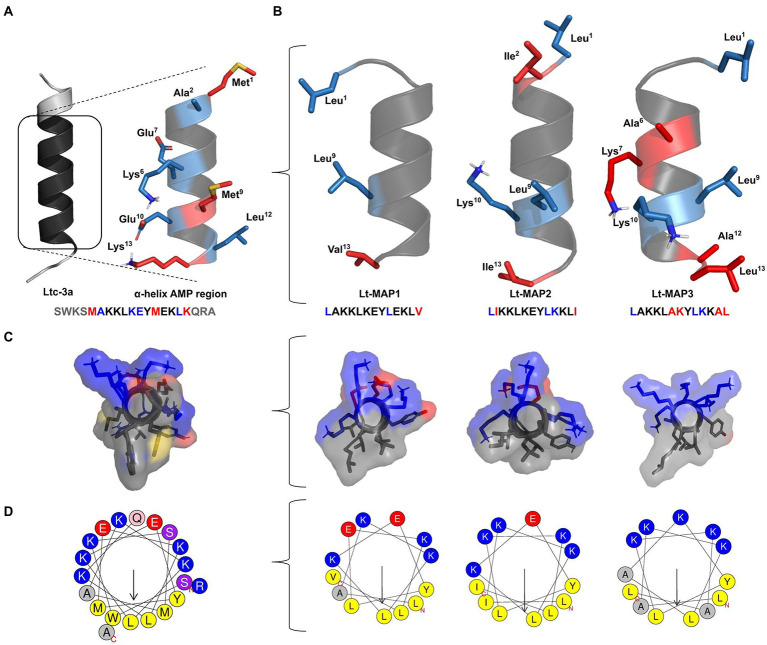
Rational design of latarcin (Ltc-3a) analog peptides. Initially, **(A)** reduction in the length of the primary sequence of the toxin; **(B)** Analogs peptides,Lt-MAP1, Lt-MAP2, and Lt-MAP3, with the three-dimensional structures and primary sequence of the AMP region represented by red: non-repeated amino acid residues in all analogs and blue: repeated amino acid residues shared between both analogs. **(C)** Front view for electrostatic surface of the analog’s peptides, and **(D)** Helix diagram comparing adjusted amphipathic and hydrophobic moment.

The rational design of the peptides was performed based on the reduction of the length of the primary sequence using the α-helix region of the AMP structure tridimensional. The punctual modifications were carried out in the sequence allowing the adjustment of the amphipathicity, charge, hydrophobicity, and hydrophobic moment of the peptides to the analogs. In addition, the helix diagram and structural model prediction were visualized demonstrating the electrostatic surface (Figure 1).

**Figure 2:**
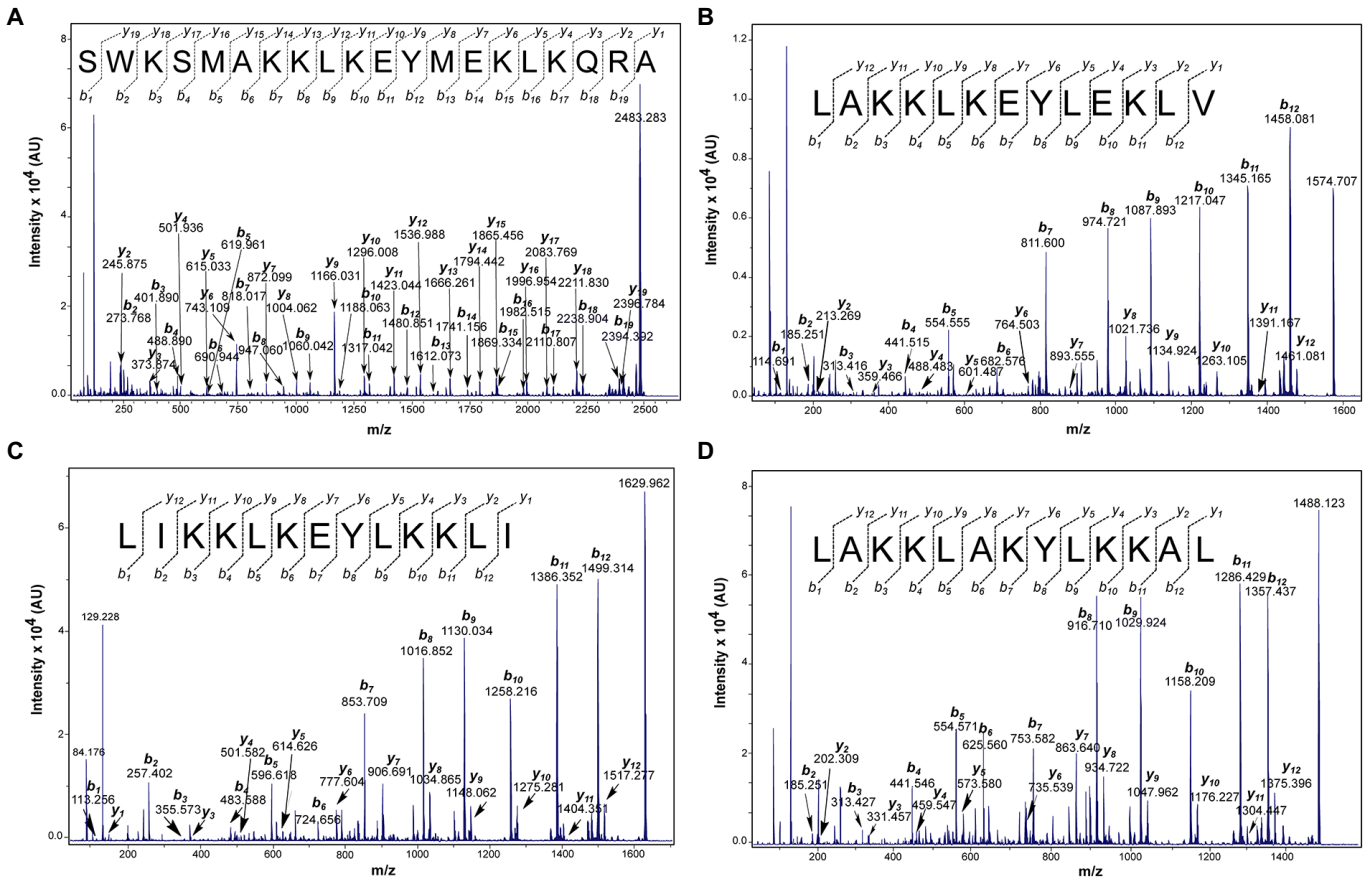
Matrix Assisted Laser Desorption Ionization Time of Flight Mass Spectrometry (MALDI-ToF MS/MS) spectra of the peptides indicating -y and -b series. **(A)** Ltc-3a, parental ion: 2483.283 m.z^−1^; **(B)** Lt-MAP1, parental ion 1574.707 m.z^−1^; **(C)** Lt-MAP2, parental ion: 1629.962 m.z^−1^; and **(D)** Lt-MAP3, parental ion: 1488.123 m.z^−1^. The data were acquired in LIFT mode and the sequences were determined manually.

All synthetic peptides were C-terminus treated as a carboxylate group, after the synthesis and purification of the peptides, a mass spectrometry technique was performed, in order to confirm the real mass of the peptides as well as their purity. The purification profiles were evaluated and all synthetic peptides demonstrated a single isolated peak majority confirming their purity above 95% (Supplementary Figure 1). The molecular mass for Ltc-3a, Lt-MAP1, Lt-MAP2, and Lt-MAP3 was calculated by electrospray ionization (ESI) mass spectrometry and presented 2483.3, 1575.2, 1630.2, and 1488.0 Da, respectively (Supplementary Figure 2). In addition, the primary sequence for each synthetic peptide was confirmed using collision-induced dissociation by mass spectrometry MALDI-ToF/ToF (Bruker, German; Figure 2).

### *In silico* molecular modeling and alignment

The analogs peptides were aligned using the ClustalW server for comparison with the model sequence, allowing the visualization of amino acid substitutions. The identity and difference were calculated for each analog, based on the alignment with parental fragment sequence, the analog peptides have an identity when compared to the sequence of parental peptide, the analogs obtained an identity of 76.9, 61.5, and 46.6% to Lt-MAP1, Lt-MAP2, and Lt-MAP3.

The predictions of the three-dimensional structure of the Ltc-3a peptide and Lt-MAPs showed that these compounds tend to assume α-helix conformation, with some analogs having a short random tail at their terminals. The Iterative Threading Assembly Refinement (I-TASSER) server, when comparing analogues structures present in the PDB database, performs a hierarchical approach to protein structure and function prediction.

Initially, there is the identification of structural models of the PDB using the LOMETS multiple chaining approach, with complete atomic models built by simulations of assembling iterative model fragments. The discoveries made by the chaining of 3D models generate the function of the BioLIP protein, generating analytical data, such as the three-dimensional models and RMSD, C-score, and Z-score (Supplementary Table 1).

### *In vitro* analysis

#### Antibacterial activity

Micro dilution tests were performed to determine the synthetic peptide capacity to reduce bacterial growth. Ltc-3a demonstrated a low activity spectrum compared to most of the tested bacteria, except for *A. baumannii, P. aeruginosa,* and *K. pneumoniae*, which MIC was 4, 128, and 128 μg.ml^−1^, respectively. Although Ltc-3a has not been demonstrated MIC for the bacteria tested here, it was possible to detect growth inhibition of 37 and 42%, respectively for *E. coli* ATCC and KPC, with the value of 128 μg.ml^−1^ for both strains (Table 2).

**Table 2:** Minimal inhibitory concentrations (MIC) and minimal biofilm inhibitory concentrations (MBIC) of latarcin-3a and its analogs Lt-MAPs, against Gram-negative and -positive bacteria strains.

Bacterial strains (Gram-negative)	MIC (μg.ml^−1^)				
	Ltc-3a	Lt-MAP1	Lt-MAP2	Lt-MAP3	Ciprofloxacin
*A. baumannii (ATCC 003321216)*	4	*>128*	8	128	128
*E. coli (ATCC 25922)*	*>128*	*>128*	*>128*	64	4
*E. coli (KPC+ 001812446)*	*>128*	*>128*	*>128*	64	128
*K. pneumoniae (ATCC 13883)*	*128*	*>128*	32	32	32
*K. pneumoniae (KPC+ 001450421)*	*>128*	*>128*	64	*>128*	128
*P. aeruginosa (KPC+ 003321199)*	*128*	*>128*	128	*>128*	64
Bacterial strains (Gram-positive)	Ltc-3a	Lt-MAP1	Lt-MAP2	Lt-MAP3	Ciprofloxacin
*P. acnes (ATCC 51277)*	*>128*	*>128*	*64*	16	*>128*
*S. aureus (ATCC 7133623)*	*>128*	*>128*	*>128*	128	64
Biofilm bacterial strains	MBIC (μg.ml^−1^)				
*A. baumannii (ATCC 003321216)*	32	*>128*	32	128	*>128*
*E. coli (ATCC 25922)*	16	128	16	64	*>128*

The results observed for the first generation of peptides demonstrated that Lt-MAP1 showed no activity against any tested bacterial strain. In contrast, the Lt-MAP2 MIC for Gram-negative bacteria was effective against *A. baumannii*, *K. pneumoniae* (ATCC 13883), *K. pneumoniae* (KPC 001450421), and *P. aeruginosa*, presenting values of 8, 32, 64, and 128 μg.ml^−1^, respectively. For Lt-MAP3, activity was observed against *A. baumannii*, *E. coli* (both strains), and *K. pneumoniae* (ATCC 13883), presenting values of 128, 64, and 32 μg.ml^−1^, respectively (Table 2).

In contrast, the MIC results for Gram-positive bacteria demonstrated that peptide Lt-MAP2 has no activity against *S. aureus*, but it was active against *P. acnes* with an MIC value of 64 μg.ml^−1^. In addition, peptide Lt-MAP3 demonstrated a MIC value of 128 and 16 μg.ml^−1^ against *S. aureus* and *P. acnes*, respectively (Table 2). MICs are confirmed by bactericidal tests and are available to *A. baumannii* and *P. aeruginosa* (Supplementary Figure 3), to *E. coli* ATCC and *E. coli* KPC (Supplementary Figure 4), to *K. pneumoniae ATCC* and *K. pneumoniae* KPC (Supplementary Figure 5), and to *S. aureus* and *P. acnes* (Supplementary Figure 6).

#### Anti-biofilm activity

**Figure 3:**
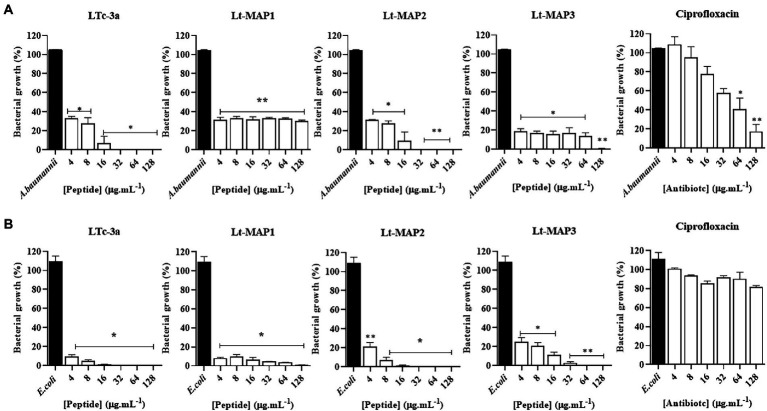
Antibiofilm activity of Ltc-3a peptides and their analogs, Lt-MAP1, Lt-MAP2, and Lt-MAP3 at 128–4 μg.ml^−1^ concentrations. **(A)** *Acinetobacter baumannii*—ATCC; **(B)** *Escherichia coli*—ATCC. Values are means ± Phase Dispersion Minimization of three repetitions. *symbol is statistically significant. (*) *p*  > 0.01 and (**) *p*  > 0.001.

Among the bacteria available, two strains were able to form biofilm for carrying out the experiments, namely *A. baumannii* and *E. coli* (ATCC 25922). For the first time in the literature, this article describes the antibiofilm activity for the parent peptide Ltc-3a, with values of 32 and 16 μg.ml^−1^, for *A. baumannii* and *E. coli*, respectively (Figure 3). For the synthetic designed peptides, Lt-MAP1 only presented activity against *E. coli* at a concentration of 128 μg.ml^−1^, and Lt-MAP2 and Lt-MAP3 were active against both strains described, with 128 and 64 μg.ml^−1^ for *A. baumannii* and *E. coli*, respectively (Table 2).

#### Hemolytic activity

For the peptides tested, the concentration and percentage of hemolysis were evaluated for Ltc-3a, presenting 80% of hemolysis in 128 μg.ml^−1^. On the other hand, analogs Lt-MAP1, Lt-MAP2, and Lt-MAP3 did not demonstrate a significant hemolysis rate (below 20% of hemolysis) between 4 and 128 μg.ml^−1^. Only the parental peptide Ltc-3a demonstrated statistically significant hemolytic activity to calculate EC_50_, which showed a value of 127 μg.ml^−1^. The hemolytic activity of each peptide is available (Supplementary Figure 8).

#### Antitumor activity

The cytotoxic activity of the peptides was evaluated at 50 μM for 72 h for initial screening. Tumor hematological lines C1498 (murine myeloid leukemia), Kasumi-1 (acute human myeloid leukemia), K562 (chronic myeloid leukemia), MOLT4 (acute lymphoblastic leukemia), Jurkat (human acute T cell leukemia), and Raji (Burkitt’s lymphoma) were used. In general, the peptides had more effect on the myeloid strains. For the first time, this article describes the anti-leukemia activity for the parent peptide Ltc-3a, which demonstrated cell viability rates of 20.5, 10 and 30% for C1498, Kasumi-1 and K562 cell lines, respectively. Among the analogs, the most promising were Lt-MAP2, obtaining a reduction in viable cells of 18% for C1498 and 5% for Kasumi-1. Lt-MAP1 and Lt-MAP3 were not selected in the cell viability test, as they did not obtain cytotoxic activity against tumor cells at the concentration tested, available in Supplementary Figure 9.

After selecting the most active peptides (Ltc-3a and Lt-MAP2) in strains C1498 and K562, concentration-response curves were performed after 24 h of treatment. From the curves, Emax and EC_50_ were calculated. The curves show a low activity in the K562 line, showing better activity in the C1498, when compared to the other strain tested; both peptides were more active at the concentration of 50 μM. The peptide Lt-MAP2 showed the highest activity (Emax) in C1498 and K562 lines, respectively, which also corresponded to the highest observed potency (EC_50_), however, when compared with the commercial antineoplastic drug, daunorubicin, was observed more efficient than the peptides, promoting a reduction the tumor cell viability at concentrations 50 and 25 μg.ml^−1^ for C1498 and K562, respectively (Supplementary Table 2).

## Discussion

Bacterial and hospital infections linked to bacterial resistance are responsible for the death of many people worldwide (Agyepong et al., 2018). There are also numerous diseases caused by disorders of the human organism itself, such as tumors (Deslouches and Di, 2017). There is a need to discover and develop new compounds with biotechnological therapeutic potential, to combat bacterial resistance as well as tumor cells.

Antimicrobial peptides are frequently and persistently studied and have proved to be excellent candidates in the combat and control of microorganisms. Many of these have a wide spectrum of biological activities, denominated promiscuous, or multi-active peptides (Silva et al., 2011). The biological activity of peptides is considered a major factor in implementing new medicines (Pfalzgraff et al., 2018).

Works using rational design of antimicrobial peptides can be a useful tool for the development of new biotechnological alternatives for the treatment of several pathologies (Chen and Lu, 2020).

In the literature, numerous works are described using rational design techniques, with modifications in the primary peptide sequence for different applicability (Lima et al., 2021). An example is the synthetic peptide Pa-MAP2, derived from the polar fish *Pleuronectes americanus*, an alanine-rich multifunctional palindromic compound that has antibacterial, antifungal, antiviral, antitumor, and antifreeze activity, with MICs at concentrations of 3.2 μM (Migliolo et al., 2016).

Another study carried out with snake-derived toxins developed two new rationally designed synthetic helical peptides, BotrAMP14 and CrotAMP14 derived from batroxicidin and crotalicidin, respectively. The redesign of the peptide sequences enabled broad-spectrum antibacterial activity against susceptible bacteria and clinical isolates at concentrations of 2–35.1 μM, acting as membrane disruptors (Oliveira et al., 2020).

Peptides from the dermaseptin family derived from anuran cutaneous secretion are one of the most studied in recent years. A study with dermaseptin-AC from *Agalychnis callidryas* designed two synthetic analogs, DRP-AC4a and DRP-AC4b, one increasing the charge and another the hydrophobicity, respectively. Both analogs obtained an increase in the spectrum and antibacterial activity and hemolytic activity reduction, when compared to the natural peptide (Gong et al., 2020).

L1G, L7a, and L1GA5K are analogs derived from mastoparan-C peptide from *Vespa crabro*. The peptides were developed to reduce hemolytic activity and maintain selectivity for pathogenic cells. The result showed activity against rifampin-resistant Gram-negative bacteria (Zhu et al., 2021). IG-13-1 and IG-13-2, analogs derived from human antimicrobial peptide LL-37, showed inhibition of biofilm formation and bacterial strains of *Streptococcus mutants* (Chen et al., 2019).

Understanding the relationship between the amino acid residue distribution and the three-dimensional structure can be the key to the development of synthetic AMPs (Migliolo et al., 2016). In general, AMPs share common characteristics, such as positive net charge and amphipathicity (Takahashi et al., 2010).

Peptide Ltc-3a is an example of these characteristics shared among peptides deposited in the APD (Wang et al., 2016). Found in the venom of the spider *L. tarabaevi*, Ltc-3a has a length of 20 aa (NH_2_-SWKSMAKKLKEYMEKLKQRA-COOH), and it is a cytolytic peptide with net charge +6, hydrophobic of 35%, hydrophobic moment of 0.575 and molecular mass of 2483.3 Da, assuming a random structure in water and an α-helix in membrane environments, which gives it selectivity for different cell types (Kozlov et al., 2006). The same peptide is also present in the scorpion venom of the genus *Centruroides* and demonstrated activity against *E. coli* and *S. aureus* (Garcia et al., 2013).

The latarcin family of peptides is characterized by a strong cationic charge (+2 to +10) and a distribution of hydrophilic and hydrophobic residues that favors amphipathicity as well as an α-helix formation in the membrane microenvironment (Kozlov et al., 2006; Dubovskii et al., 2015). The analogs were designed based on the ^5^MAKKLKEYMEKLK^17^ region, seeking to maintain similarity in the primary sequence and in the conformation.

Amino acids such as methionine and alanine were replaced by leucines and isoleucines, residues with an aliphatic side chain. One of the objectives for rational design is increase of chance to construct a molecule with pharmacological potential in the future. For this the Ltc-3a toxin, was modified to increase hydrophobicity based on the Eisenberg scale, using amino acid residues that contained only carbon atoms in their side chain and favored the formation of an α-helix structure (Eisenberg et al., 1982). Furthermore, the distribution of leucines along a hydrophobic face was a crucial decision, because studies indicate that the presence of four leucines distributed on the hydrophobic face of the latarcin (Ltc-1) from *L. tarabaevi* peptide plays a fundamental role in its insertion in membrane mimetic models and also for its antiviral activity (Dubovskii et al., 2008; Rothan et al., 2014). Aliphatic nonpolar agents such as alanines and leucines favor the formation of α-helices; in addition, the leucine and isoleucine side chains can be more easily anchored in the hydrophobic portion of the membrane (Migliolo et al., 2016).

For example, the peptide latarcin-1 (NH_2_-SMWSGMWRR KLKKLRNALKKKLKGE-COOH) also present in the spider toxin *L. tarabaevi* has in its primary sequence four leucines in positions 11, 14, 18, and 22, which contribute to the formation of an amphipathic α-helix structure in membrane environments. In addition, it has a hydrophobic N-terminal composed of two aromatic residues (Trp^3^ and Trp^7^) of high hydrophobic potential when compared to the aliphatic nonpolar residues. These residues promote the α-helical stability of the peptide latarcin-1 in a biphasic environment, such as cell membrane (Dubovskii et al., 2008).

Changes in the primary sequence of a peptide caused by adding non-polar aliphatic and/ or aromatic residues result in a direct increase in hydrophobicity potential (Chen and Lu, 2020). This factor should not be very high due to the greater probability of interaction with zwitterionic phospholipids, common in eukaryotic cells, resulting in an increase in cytotoxic and hemolytic activity (Tossi et al., 2000).

Lt-MAP1 and Lt-MAP2 showed greater hydrophobicity when compared to the parent peptide. Although both analogs have the same hydrophobicity percentage, only Lt-MAP2 showed antimicrobial activity. Thus, we can see that changes in hydrophobicity alone are not sufficient to optimize the antibacterial activity of the projected peptides, since their primary sequences have a high identity with latarcin-3a.

However, the analog Lt-MAP3 showed even greater hydrophobicity, standing out even more in antibacterial activity, but we must consider the charge factor, which is higher when compared to Lt-MAP1 and Lt-MAP2.

The net charge is also an important factor and contributes to the antimicrobial activity of the peptides. Takahasshi and co-workers stated that there is an exaggerated frequency of cationic antimicrobial peptides, and this characteristic is common due to the presence of lysine and arginine residues, while anionic amino acids, such as aspartate and glutamate, are not common (Takahashi et al., 2010).

Antimicrobial peptide studies have shown that an increase in net charge reflects cytotoxic activity and loss of cellular selectivity toward bacterial membrane components (Takahashi et al., 2010; Silva et al., 2022). In addition, amphipathic deformations, resulting from reduced hydrophobicity, contribute to selectivity and interaction with anionic membranes of pathogens and tumor cells (Gagnon et al., 2017; Mas et al., 2017).

The analogs, Lt-MAP1 and Lt-MAP2, have a lower cationic charge than latarcin-3a. Lt-MAP1 has two glutamic acid residues (Glu^7^ and Glu^10^) and Lt-MAP2 has one (Glu^7^), both positioned on the α-helix hydrophilic face. The presence of anionic residues implies the theoretical neutralization of cationic residues resulting from the subtraction of charges.

Seeking to maintain the identity between the analogs and the parent, it was proposed that the glutamic acids remain in Lt-MAP1, resulting in a +2 charge, while in Lt-MAP2 there was a substitution of Glu^10^ for a lysine, allowing an increase in the charge to +4, but still maintaining a strong identity of 50 and 40% when compared to the native.

Keeping the acid residues in Lt-MAP1 resulted in low antibacterial activity, with no minimum inhibitory concentration determined against bacteria tested. However, as observed in Lt-MAP2, the increase in charge to +4 may have favored peptide-membrane interactions, resulting in cell death.

Lt-MAP3 differs from the other analogs and from the native in that it does not present acid residues, with presence of just lysines. This has shown a noticeable improvement in the antibacterial activity of both peptides, Lt-MAP1 and Lt-MAP2.

Gomesin can be considered an example that peptides can act at lower molar concentrations than antibiotics and their advantages to bacterial resistance (Silva et al., 2000). Studies show the activity of gomesin against Gram-negative and -positive bacteria with MICs ranging from 0.4 to 1.2 μM, while the antibiotic amphotericin-B did not obtain antimicrobial activity against strains of *Micrococcus luteus*, *S. aureus*, *E. coli*, and *P. aeruginosa* (Ayroza et al., 2012).

Another study carried out with a rational design of synthetic peptides derived from temporin-PTa, a toxin from the anuran *Hylarana picturata*, also proved to be better than the antibiotic, when compared to its micromolar concentration in a range of 2.8–98 μM and broad spectrum of antibacterial activity, such as *E. coli*, *K. pneumoniae*, *A. baumannii*, and Methicillin-resistant *S. aureus* (Silva et al., 2022).

Regarding antitumor activity, recent studies have confirmed the effectiveness of these compounds in combating tumor cells. Tumor cells usually show an increase in the levels of phosphatidylserine on the membrane surface when compared to normal cells, which can result in an interaction target for cationic amphipathic peptides, making these compounds an effective and selective source of antitumor agents and that have some mechanism of resistance (Deslouches and Di, 2017).

The discovery of compounds with antibiofilm activity is recent and few peptides derived from spiders are described with this type of functionality, according to the APD database, many have activity against planktonic bacteria, however further studies are needed (Wang et al., 2016). Among these, we can mention Lycosin-II, present in the toxin of the spider *Lycosa singoriensis*, active against biofilms of multidrug-resistant bacteria such as oxacillin-resistant *S. aureus* and meropenem-resistant *P. aeruginosa* (Oh et al., 2022).

The latarcins peptide family does not describe this type of activity for any of the peptides present in the APD database (Dubovskii et al., 2015), and the result of this article is an unprecedented description for Ltc-3a, active against *A. baumannii* and *E. coli* strains. The other synthetic peptides also showed promise for combating biofilm-forming strains, revealing a new perspective for approaching antibiofilm tests with peptides derived from spiders.

In the literature, peptides derived from spiders that have antitumor activity are poorly described. An example is gomesin, which is a cationic and multi-active peptide with a β-sheet structure, a net charge +6 and 18 amino acid residues (NH_2_-QCRRLCYKQRCVTYCRGR-COOH). It has activity against several types of bacteria and antitumor activity, derived from the hemolymph of the spider *Acanthoscurria gomesiana* (Silva et al., 2000).

More recent studies dealing with peptides derived from the spider *L. tarabaevi* toxin demonstrated that Latarcin-2a (Ltc-2a), with a net charge of +9 and 26 amino acid residues (NH_2_-GLFGKLIKKFGRKAISYAVKKARGKH-COOH) chain, with has antitumor activity against the erythroleukemia cells of the K562 strain EC_50_ in 3.3 μM and in erythrocytes EC_50_ in 3.4 μM (Vorontsova et al., 2011).

When we compare the synthetic peptides Lt-MAPs with Ltc-3a and Ltc-2a, we can observe that in this case, the natural peptides have better activity and greater spectrum of action in different strains, however, both have high toxicity for erythrocytes, while the analogs, mainly Lt-MAP2 was shown to have a higher affinity to C-1498 and K562 strains and low toxicity to normal red blood cells.

In addition, studies have shown antitumor activity in Ltc-1 against tumor cell lines (HEPG2 and MCF-7), inhibiting cell proliferation in a dose-dependent manner, reaching higher inhibitory potential at a concentration of approximately 200 μM (Rothan et al., 2015). When comparing Ltc-1 with Ltc-3a, we observe that the concentration to obtain max activity is lower, 156.4 and 148.8 μg.ml^−1^ (51 and 74.4 μM) for the K562 and C1498 strains, while for the Lt-MAP2 analog, the values are similar to the parental with 129.6 and 177.4 μg.mL^−1, respectively,^ (64.8 and 88.7 μM).

The commercial antineoplastic drug daunorubicin was more effective than Ltc-3a and its analog Lt-MAP2 on concentration-response curves, promoting inhibition of cell viability at lower concentrations than peptides. However, drugs belonging to the anthracycline class, such as daunorubicin and doxorubicin, have a number of proven side effects to the body, as cardiotoxicity (Cruz et al., 2016).

In addition, anthracyclines, which act as enzyme topoisomerase II inhibitors, form free radicals inside the cell, that result in cell death by apoptosis or necrosis (McGowan et al., 2017). In contrast, a study carried out with doxorubicin demonstrated the hemolytic potential that anthracyclines can cause in blood cells, showing that despite its efficiency in the treatment of tumor cells, it can still cause side effects to patients (Santos et al., 2018).

The division between hydrophilic and hydrophobic residues positioned along the α-helix structure gives the peptides an amphipathic character, enabling interaction with pathogenic membranes (Migliolo et al., 2016). Variations in these parameters interfere with cell selectivity and can simultaneously increase antibacterial and cytotoxic activities (Takahashi et al., 2010).

The hydrophobic moment, acting in conjunction with hydrophobicity and the cationic liquid charge, are parameters that can confer the formation of α-helical conformations in membrane environments to which they are attracted by electrostatic interactions, enabling an improvement in antimicrobial activity (Migliolo et al., 2016).

Latarcin-3a interacts easily with the membranes, due to its α-helical structure, which has a well-defined amphipathicity, with a hydrophobic moment of 0.575. The analogs remained similar to the native, showing amphipathic character; however, there was an increase in the hydrophobic moment of 0.740, 0.839, and 0.701, respectively. The predictions of the three-dimensional structure of the analogs are within the reliability values of the structural homology, and all analogs can assume the α-helix conformation.

When carrying out purification and identification studies for the peptides present in the toxin, Kozlov et al. (2006) carried out an alignment study between the sequences of the latarcins found in the venom of the *L. tarabaevi* spider. Thus, we can suggest that the presence of residues such as tryptophan, phenylalanine, leucine, lysine, and tyrosine are essential for preserving latarcin’s biological activities.

Rational design techniques that promote size reduction and cytotoxicity for the development of promiscuous peptides with different interaction targets can affect large-scale production. Many natural peptides are not only selective for pathogens, but also for normal human cells, for example, oxyopinin-1 from the spider *Oxyopes kitabensis*, which has antibacterial activity and promotes blood cell hemolysis (Garcia et al., 2013).

In the literature, the cytotoxicity against mammalian cells that peptides derived from spider toxin present is well described (Gautam et al., 2014; Wang et al., 2016). Some of these peptides have high toxicity, among them we can mention oxyopinin-4 with EC_50_ at 7 μM (Dubovskii et al., 2011). Other peptides from the latarcins family, such as Ltc-1, Ltc-2a, and Ltc-5 also have strong toxicity to human and murine erythrocytes (Kozlov et al., 2006; Dubovskii et al., 2015; Wang and Wang, 2016).

The rate of hemolysis and cytotoxicity is a crucial factor in the development of new synthetic peptides, being of great importance in the removal or reduction of this characteristic, when the objective is to develop new drugs, which can be achieved through modifications/substitutions of amino acids along the primary sequence, altering parameters of physicochemical characteristics such as net charge and hydrophobicity (Takahashi et al., 2010; Wang et al., 2021).

The analogs developed in this work did not demonstrate high rates of hemolysis, being below 20% at the highest concentrations tested, as observed in the results, in contrast to the parental peptide Ltc-3a, where its cytotoxicity and hemolytic potential are described (Kozlov et al., 2006).

When comparing the amino acid composition of the developed peptides, it was possible to observe interesting differences between them, which are reflected in their biological activity. Among Lt-MAP1 and Lt-MAP2, we could observe some amino acid residues replacements as Ala^2^, Glu^10^, and Val^13^ in Lt-MAP1 by Ile^2^, Lys^10^, and Ile^13^ in Lt-MAP2. Those changes promote an increase of positive charge, but mainly of the hydrophobicity thus as hydrophobic moment, which allowed Lt-MAP1 to be selective to *E. coli* biofilms and in addition, hemolytic. In contrast, Lt-MAP2 presented antibacterial, anti-biofilm, and antitumor activities and not hemolytic activity.

Ma and collaborators evaluated the leucine repetition for LRR1 and LRR2, synthetic peptides developed from a *de novo* design. Their results demonstrated that these residues can favor the peptide anchoring on mimetic cell membranes as a result of hydrophobic increase (Ma et al., 2013).

Furthermore, the residues replacements for Lt-MAP1 and Lt-MAP3 were Lys^6^, Glu^7^, Glu^10^, Leu^12^, and Val^13^ in Lt-MAP1 by Ala^6^, Lys^7^, Lys^10^, Ala^12^, and Leu^13^ in Lt-MAP3. That also promoted an increase of positive charge but maintains both, hydrophobicity and hydrophobic moment. Lt-MAP3 presented antibacterial activity against Gram-negative and -positive bacteria and biofilm, but no antitumoral activity nor hemolysis. Another observation was that alanine promoted an improvement in the amphipathicity due to favoring of the α-helices formation.

Park and collaborators showed that the substitution of alanine or lysine within the leucine zipper motif in the pseudin-2 sequence suggested an increase in the selectivity of the microorganisms’ membranes (Park et al., 2020). In addition, alanine-rich peptides are more likely to form a helix (Zhuang et al., 2021).

Lastly, the difference between Lt-MAP2 and Lt-MAP3 amino acid residue replacements were for Ile^2^, Lys^6^, Glu^7^, Leu^12^, and Ile^13^ in Lt-MAP2 by Ala^2^, Ala^6^, Lys^7^, Ala^12^, and Leu^13^ in Lt-MAP3. That promoted an increase of positive charge, decreasing the hydrophobicity and the hydrophobic moment, but the latter smoothly. In addition, it was also observed that alanines have an important role in the amphipathicity. These modifications resulted in the evident multifunctionality for Lt-MAP2.

The substitution of glutamic acid by lysine favored an increase of charge, directly implicating cell selectivity in the synthetic peptides S3E3 and S3E3A, developed from the S3 peptide derived from the lipopolysaccharide-binding site of factor C protein, present in horseshoe crab’s hemolymph (Sepahi et al., 2020).

## Conclusion

The peptide analogs developed to provide better antimicrobial activity when compared to the parent latarcin-3a. The best ones were Lt-MAP2, effective against bacteria and tumor cells, and Lt-MAP3, active against resistant and non-resistant bacterial strains. Antimicrobial promiscuous peptides have proven to be excellent candidates for the development of alternative bioactive compounds to combat and control bacteria and tumor cell lines.

## Data availability statement

The original contributions presented in the study are included in the article/Supplementary material; further inquiries can be directed to the corresponding author.

## Author contributions

LMo and LMi designed, revised, and edited the final version of the manuscript. LMo, TP, TA, HT, CL, and EP-G performed the *in vitro* experiments. PS and BF analyzed the data. RC performed the sequencing of synthetic peptides by mass spectrometry. All authors contributed to the article and approved the submitted version.

## Funding

This work was supported by CAPES (Coordenação de Aperfeiçoamento de Pessoas de Nível Superior), FUNDECT (Fundação de Apoio ao Desenvolvimento da Educação, Ciência e Tecnologia do Estado de Mato Grosso do Sul), and CNPq (Conselho Nacional de Desenvolvimento Científico e Tecnológico) research funding agencies.

## Conflict of interest

The authors declare that the research was conducted in the absence of any commercial or financial relationships that could be construed as a potential conflict of interest.

## Publisher’s note

All claims expressed in this article are solely those of the authors and do not necessarily represent those of their affiliated organizations, or those of the publisher, the editors and the reviewers. Any product that may be evaluated in this article, or claim that may be made by its manufacturer, is not guaranteed or endorsed by the publisher.

## Footnotes

^1^ https://www.genome.jp/tools-bin/clustalw

^2^ https://zhanglab.ccmb.med.umich.edu/I-TASSER/

## Abbreviations

I-TASSER: Iterative threading ASSEmbly refinement
KPC: *Klebsiella pneumoniae* cabapenemase
Lt-MAPs: *Lachesana tarabaevi*-multi-active peptides
RMSD: Root-mean-square deviation.
