# Supplementary figures and images for: First generation of multifunctional peptides derived from latarcin-3a from Lachesana tarabaevi spider toxin
Source: Front Microbiol. 2022 Sep 21;13:965621. doi: 10.3389/fmicb.2022.965621 (PMC9532841; doi:10.3389/fmicb.2022.965621)

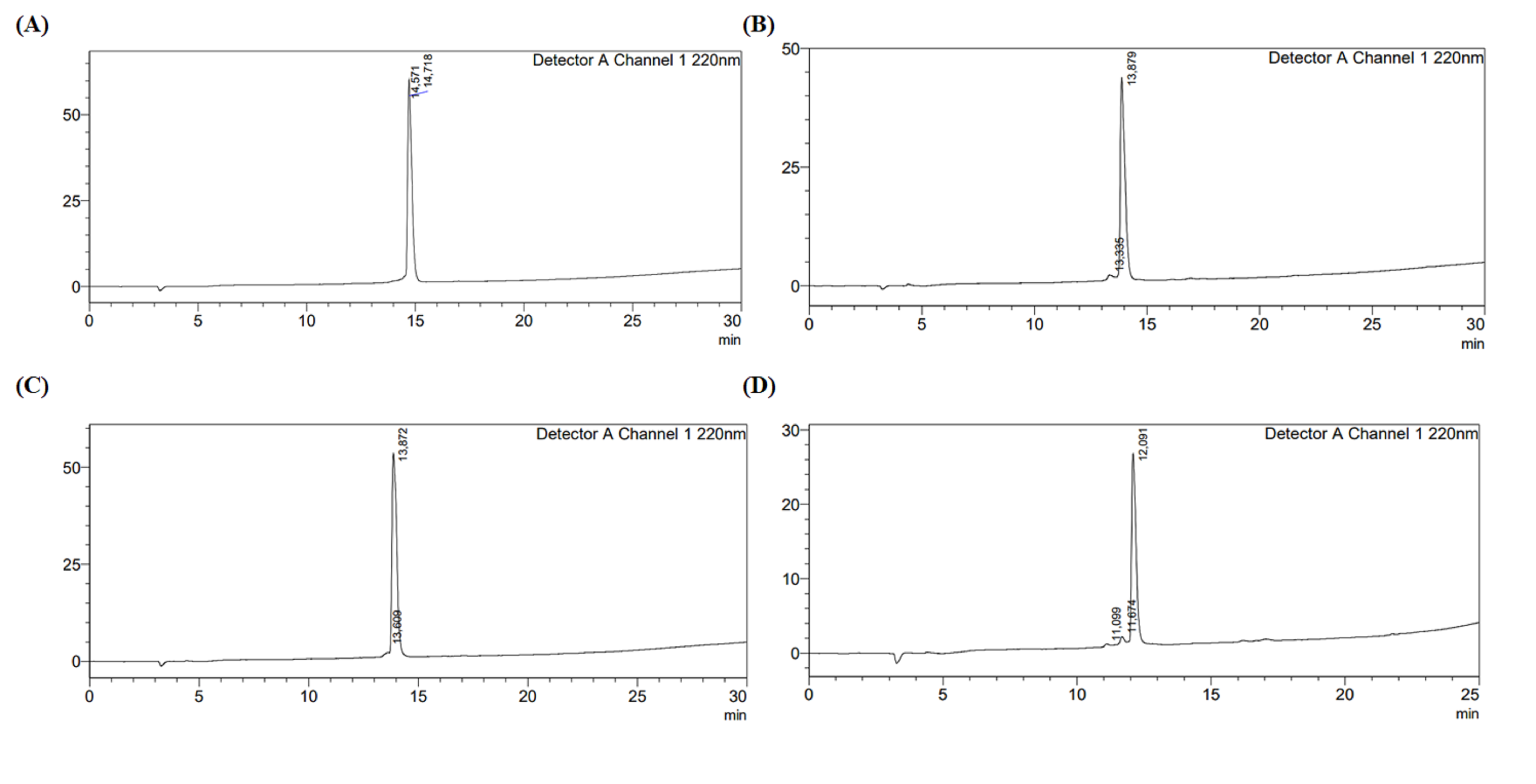

Supplement: Supplementary file 1 [file Image_1.TIF]

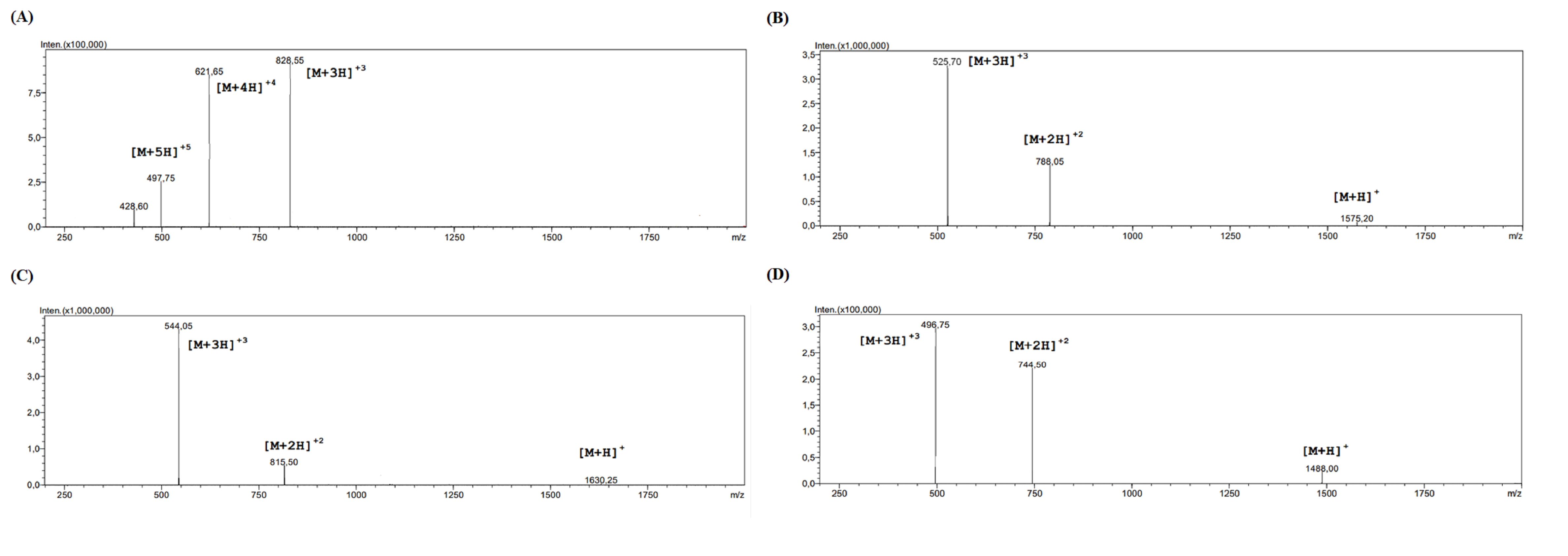

Supplement: Supplementary file 2 [file Image_2.TIF]

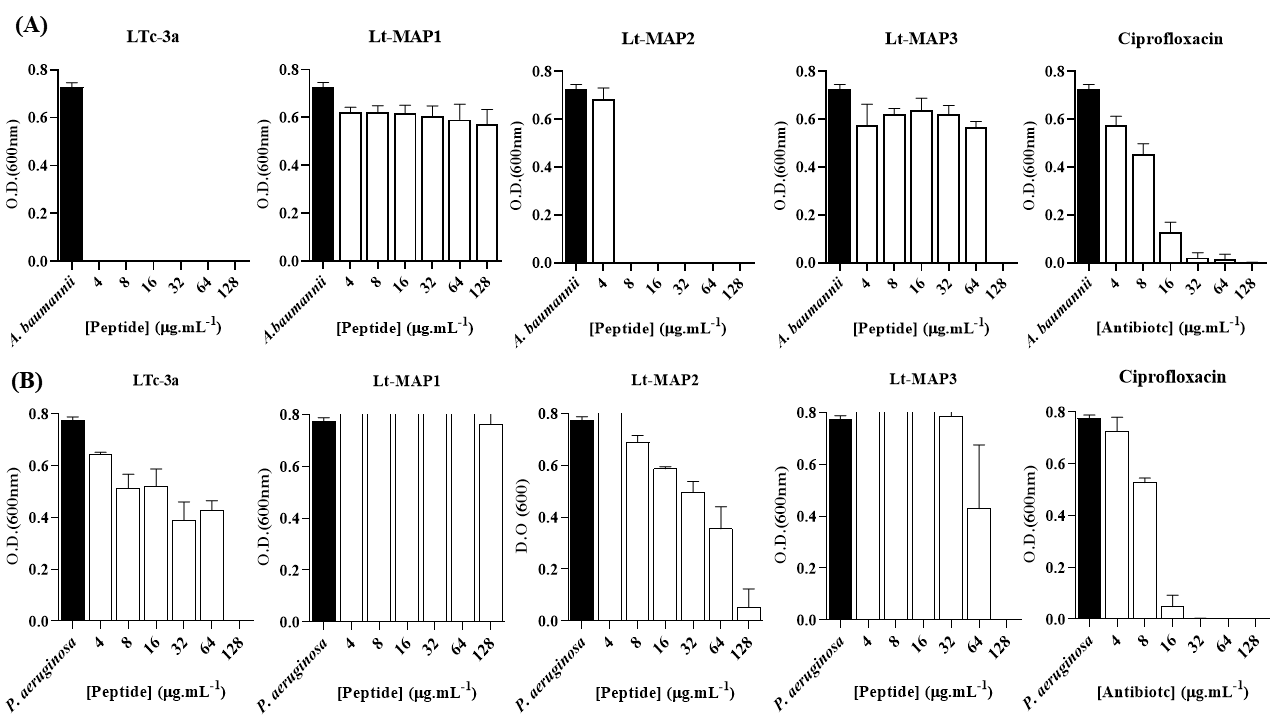

Supplement: Supplementary file 3 [file Image_3.TIF]

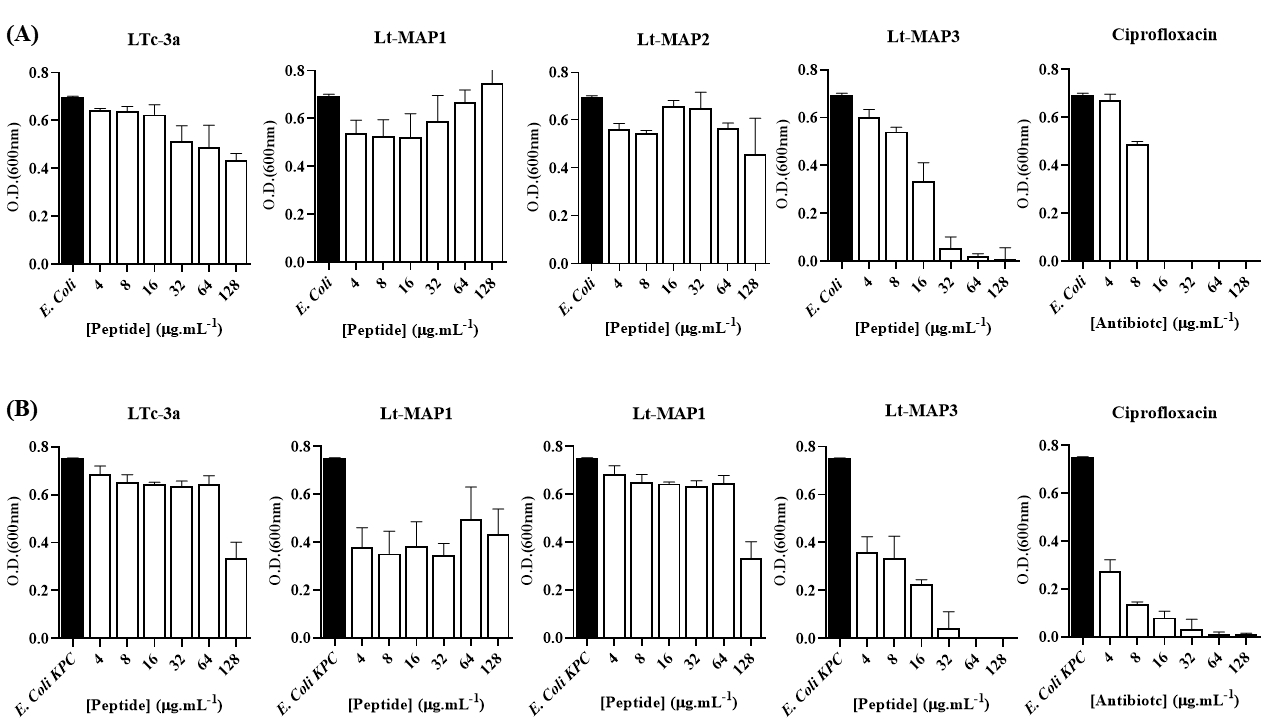

Supplement: Supplementary file 4 [file Image_4.TIF]

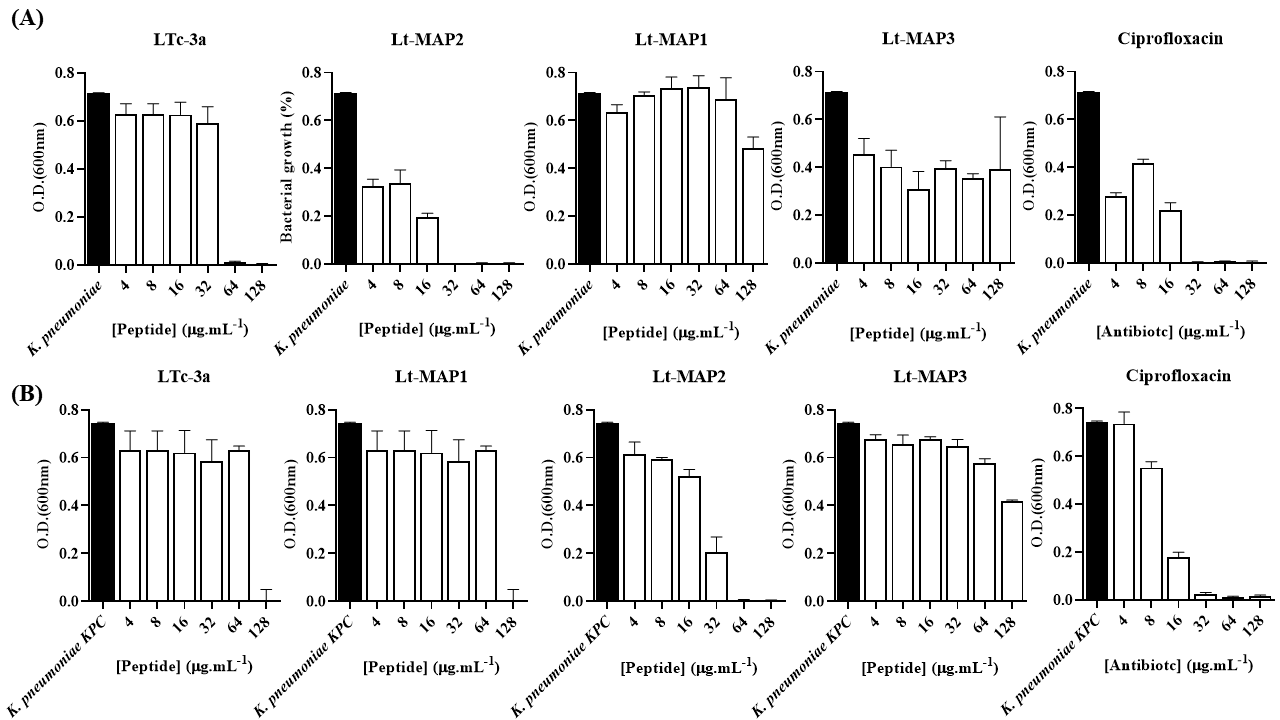

Supplement: Supplementary file 5 [file Image_5.TIF]

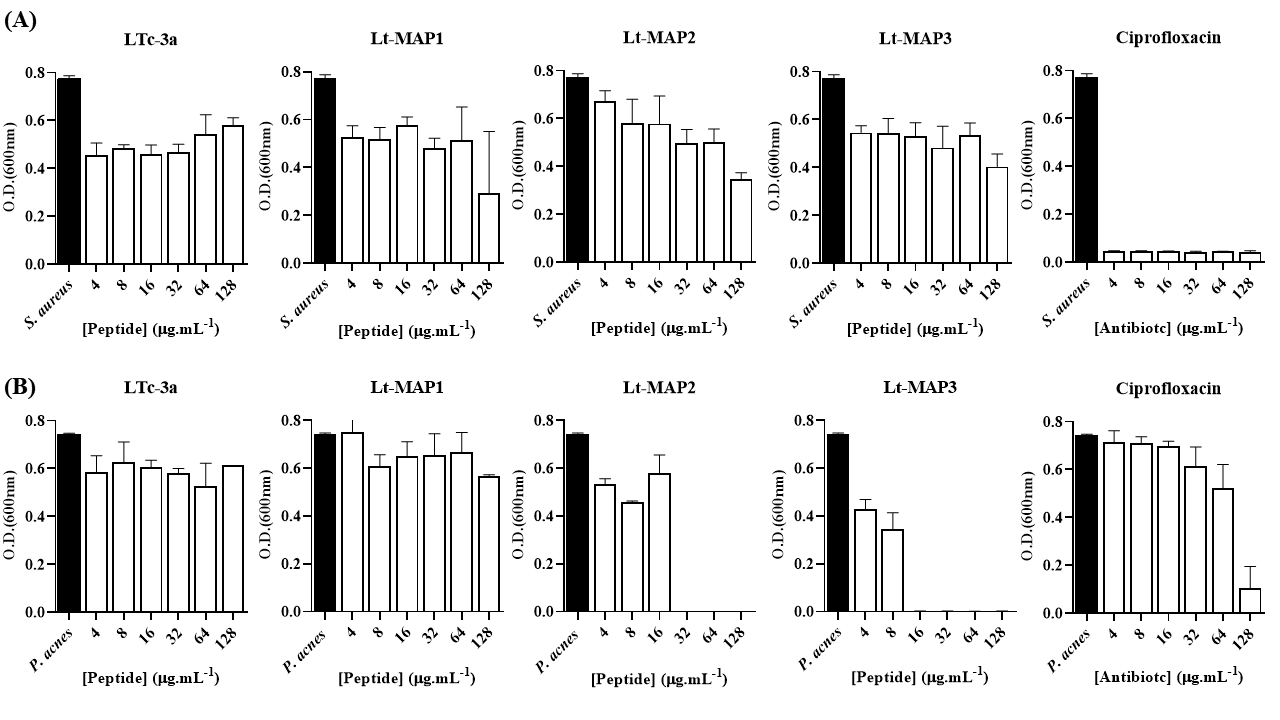

Supplement: Supplementary file 6 [file Image_6.TIF]

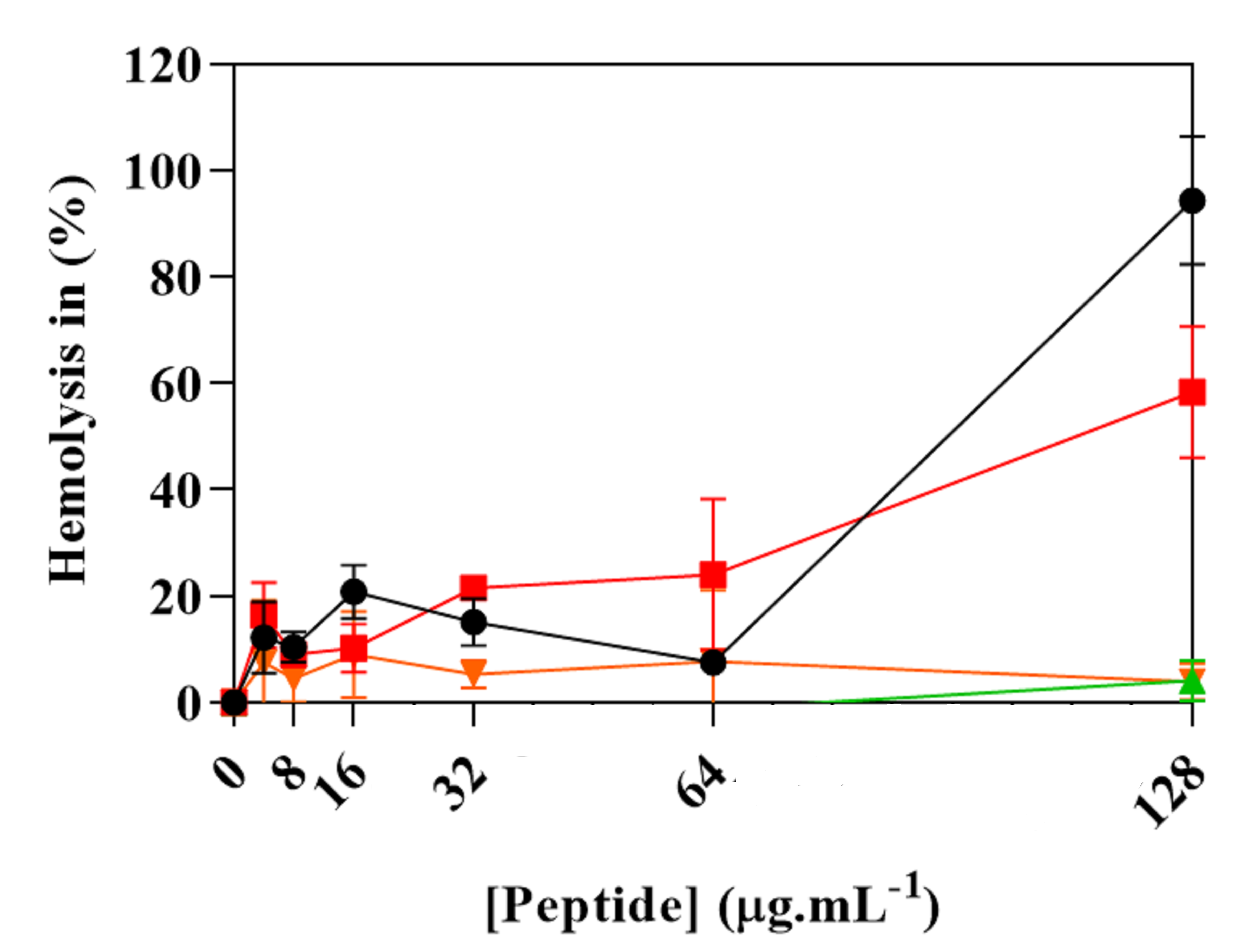

Supplement: Supplementary file 7 [file Image_7.TIF]

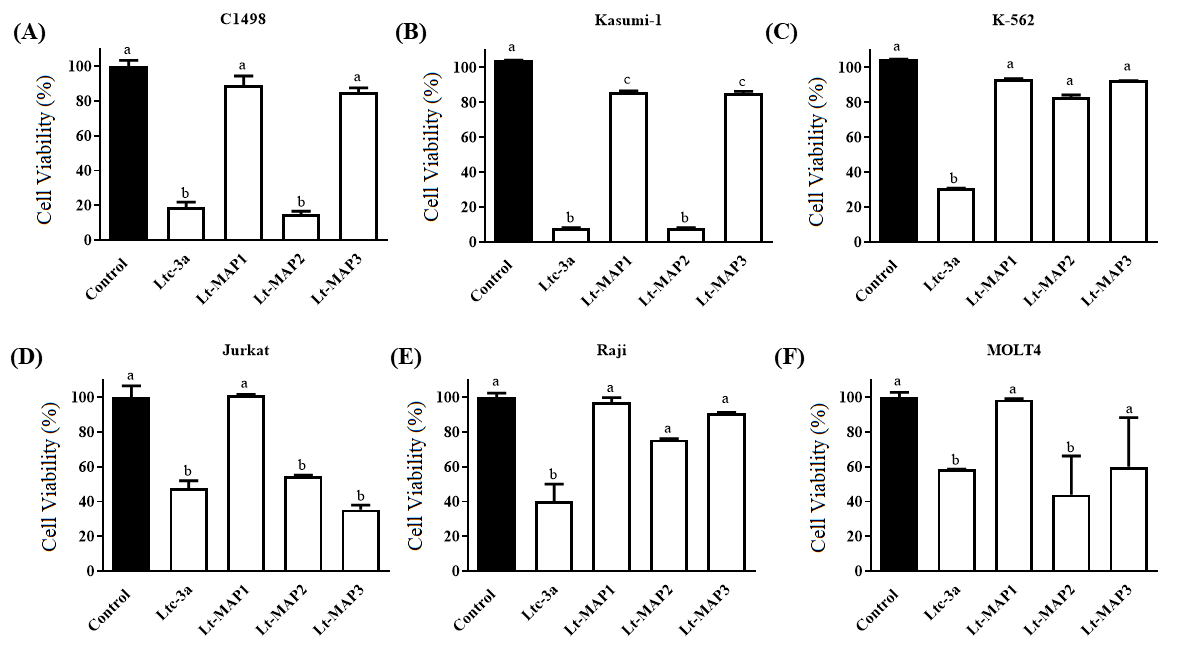

Supplement: Supplementary file 8 [file Image_8.TIF]
