# Supplementary material for: First generation of multifunctional peptides derived from latarcin-3a from Lachesana tarabaevi spider toxin
Source: Front Microbiol. 2022 Sep 21;13:965621. doi: 10.3389/fmicb.2022.965621 (PMC9532841; doi:10.3389/fmicb.2022.965621)
Supplement: Supplementary file 9 [file Data_Sheet_1.DOCX]

***Supplementary Material***

# Supplementary Figures and Tables

# Supplementary Figure 1. Profile purification by reversed-phase high performance liquid chromatography (RP-HPLC) to >95% purity for (A) Ltc-3a, (B) Lt-MAP1, (C) Lt-MAP2 and (D) Lt-MAP3 peptides.

# Supplementary Figure 2. ESI profile representing monoisotopic and multiple charges of each synthetic peptide ionized. (A) Ltc-3a, (B) Lt-MAP1, (C) Lt-MAP2 and (D) Lt-MAP3.

# Supplementary Figure 3. Antibacterial activity of Ltc-3a peptides and their analogues, Lt-MAP1, Lt-MAP2 and Lt-MAP3 at 128 to 4 µg.mL^-1^ concentrations. (A) *A. baumannii* - ATCC; (B) *P. aeruginosa* - KPC + .

# Supplementary Figure 4. Antibacterial activity of Ltc-3a peptides and their analogues, Lt-MAP1, Lt-MAP2 and Lt-MAP3 at 128 to 4 µg.mL^-1^ concentrations. (A) *E. coli* - ATCC; (B) *E. coli* - KPC + .

# Supplementary Figure 5. Antibacterial activity of Ltc-3a peptides and their analogues, Lt-MAP1, Lt-MAP2 and Lt-MAP3 at 128 to 4 µg.mL^-1^ concentrations. (A) *K. pneumoniae* - *ATCC;* (B) *K. pneumoniae* - *KPC^+^*.

# Supplementary Figure 6. Antibacterial activity of Ltc-3a peptides and their analogues, Lt-MAP1, Lt-MAP2 and Lt-MAP3 at 128 to 4 µg.mL^-1^ concentrations. (A) *S. aureus* - *ATCC;* (B) *P. acnes - ATCC*.

# Supplementary Figure 7. Evaluation of the hemolytic activity of the peptides, Ltc-3a (red), Lt-MAP1 (green), Lt-MAP2 (orange), Lt-MAP3 (blue) at 128 to 4 µg.mL^-1^ concentrations.

# Supplementary Figure 8. Percentage cell viability of peptide screening with concentration of 50 µM (Ltc-3a, Lt-MAP1, Lt-MAP2 and Lt-MAP3) in different leukemia cell models, being: (A) C1498, (B) Kasumi-1, (C) K-562, (D) Jurkat, (E) Raji and (F) MOLT4, all normalized by unstimulated sample. Equal letters do not present statistical differences.

**Supplementary Table 1:** Percentage of identity and similarity of the analog’s peptides (Lt-MAP1, Lt-MAP2 and Lt-MAP3) in relation to the parental sequence and the reliability values of the prediction of the secondary structure performed by comparative modeling of the threading type by the server I-TASSER. *****: Value unavailable for parental string.

| **Peptide** | **C-Score** | **RMSD** | **TM-Score** | **Z-Score** |
| --- | --- | --- | --- | --- |
| **Ltc-3a** | **-0.02** | **0.1 ± 0.1** | **0.71 ± 0.12** | **-1.98** |
| **Lt-MAP1** | **0.14** | **0.6 ± 0.6** | **0.70 ± 0.12** | **-1.8** |
| **Lt-MAP2** | **0.02** | **0.5 ± 0.5** | **0.72 ± 0.11** | **-2.04** |
| **Lt-MAP3** | **-0.17** | **0.6 ± 0.6** | **0.69 ± 0.12** | **-1.37** |

# Supplementary Table 2. Values of the Emax (%) and EC_50_ (µg.mL^-1^) of the peptides selected in the screening using antitumor cells and rate of hemolysis in µg.mL^-1^. *nd: activity not obtained in screening for antitumor activity.*

| **Peptide** | **K562** | | **C1498** | | **Erythrocytes** | |
| --- | --- | --- | --- | --- | --- | --- |
|  | **Emax (%)** | **EC_50_ (**µ**g.mL^-1^)** | **Emax (%)** | **EC_50_ (**µ**g.mL^-1^)** | **Hemolysis (%)** | µ**g.mL^-1^** |
| **Ltc-3a** | 156.4 ± 4.1 | 78,2 ± 1.5 | 148.8 ± 0.2 | 76,9 ± 1 | 80 | 128 |
| **Lt-MAP1** | ***nd*** | ***nd*** | ***nd*** | ***nd*** | 49 | 128 |
| **Lt-MAP2** | 129.6 ± 4.9 | 44 ± 3 | 177.4 ± 1.2 | 43,9 ± 0.5 | 3 | 128 |
| **Lt-MAP3** | ***nd*** | ***nd*** | ***nd*** | ***nd*** | 7 | 128 |
| **Daunorrubicin** | 50 ± 4.5 | 1,5 ± 0.12 | 25 ± 0.5 | 0.003 ± 0.5 | - | - |
